# Safety and long-lasting immunity of the combined administration of a modified-live virus vaccine against *porcine reproductive and respiratory syndrome virus 1* and an inactivated vaccine against *porcine parvovirus* and *Erysipelothrix rhusiopathiae* in breeding pigs

**DOI:** 10.1186/s40813-019-0118-9

**Published:** 2019-04-25

**Authors:** Almudena Sánchez-Matamoros, Agustí Camprodon, Jaime Maldonado, Rafael Pedrazuela, Joel Miranda

**Affiliations:** HIPRA, Amer (Girona), Spain

**Keywords:** *Porcine reproductive and respiratory syndrome virus*, *Porcine parvovirus*, *Erysipelothrix rhusiopathiae*, Combined administration of vaccines, Vaccine schedule, Cell-mediated immunity, Neutralizing antibodies

## Abstract

**Background:**

In the field, vaccination schedules based on modified-live virus (MLV) vaccines administered twice in gilts and every three to four months in sows are commonly used to immunize breeding herds against *porcine reproductive and respiratory virus* (PRRSV). Breeding sows are repeatedly vaccinated against several other agents. Thus, the combined administration of vaccines for their simultaneous use can simplify such complex immunization schedules. Here, we evaluated the safety and long-term immunity of the authorized combined administration of a PRRSV MLV vaccine and an inactivated vaccine against *porcine parvovirus* (PPV) and *Erysipelothrix rhusiopathiae* for their simultaneous use.

Six-month-old naïve healthy gilts were vaccinated at day 0 and revaccinated at days 21 and 147, mimicking the abovementioned vaccination schedule. Systemic and local reactions, as well as body temperature, were measured. The excretion of PRRSV1 MLV was evaluated in oral fluids. Humoral responses against the three antigens were measured by ELISA. For PRRSV, homologous neutralizing antibodies (NAs) and homologous and heterologous cell-mediated immunity (CMI) were also assessed.

**Results:**

The combined administration of the tested vaccines, applied according to the manufacturer’s instructions, was safe based on all evaluated parameters. Overall, we detected antibodies against PPV and PRRSV in all vaccinated pigs already after the first vaccination, whereas antibodies against *E. rhusiopathiae* were observed in all animals after revaccination. After subsequent revaccinations, we observed boosts for the humoral response for PPV at days 28 and 154 and at day 154 for *E. rhusiopathiae*. No boosts were detected during the experiment by PRRSV ELISA. In all vaccinated animals, homologous NAs against MLV were already detected before revaccination (day 21). After revaccination, there was a boost with mean titres of homologous NAs remaining constant thereafter. Concerning CMI, PRRSV-specific IFN-γ-secreting cells were already detected at day 21 for all evaluated strains and we observed boosts for all PRRSV1 strains after revaccination and recall revaccination.

**Conclusions:**

We showed that the combined administration of tested vaccines described here using a vaccination schedule against PRRSV commonly implemented for breeding pigs in the field is safe and induces long-lasting humoral and cellular immunity against PRRSV, PPV, and *E. rhusiopathiae.*

## Background

Since its emergence in the 1980s, porcine reproductive and respiratory syndrome (PRRS) has been considered to be one of the costliest global diseases of swine [1, 2]. Recently, a European study estimated that annual losses per farm were between € 75,724 and € 650,090, depending on the severity of the disease and the affected stages [3]. Losses associated with instability, an endemic situation in which piglets are born with asymptomatic viremia due to vertical transmission, appear to also be substantial.

The main tools for controlling PRRS virus (PRRSV) are herd monitoring by the detection of antibodies and viral circulation, the management of pig flow, biosecurity, and vaccination [4]. Knowledge of whether viremic piglets are being born is paramount to determining the best control measures to apply and the categorization of farms according to their PRRSV status [4]. When vertical transmission occurs, control measures must first be applied to breeders. Thus, robust immunization of gilts and an adequate vaccination protocol that aims to maintain immunity throughout the reproductive life of the sow are crucial. Proper PRRSV immune conditioning not only helps to reduce the risk of abortions, mortinatality, and other reproductive disorders related to PRRSV infection, but also to homogenize the immunological status of the breeding herd, reducing the chance of vertical transmission to piglets [5].

Currently, one of the most common PRRSV vaccination schedules used in the field is based on modified-live virus (MLV) administration: gilts are vaccinated and revaccinated two to three weeks apart before introducing them into the breeding herd, whereas sows are vaccinated every three to four months to maintain immunity [6]. Although schedules based on repeated immunization with an MLV vaccine have been widely implemented, few studies have evaluated the long-term immunity afforded by such schedules [7–9].

In a breeding herd, a typical immunization schedule usually includes several vaccines against PRRSV, *porcine parvovirus* (PPV), *Aujeszky’s disease virus, porcine circovirus 2, swine influenza virus, E. coli*, and *Erysipelothrix rhusiopathiae,* to name just a few, which are often administered following complex schedules, resulting in high vaccination pressure. Under such conditions, combined administration of vaccines simplifies immunization schedules by combining multiple antigens into a single injection. This approach improves both animal welfare and the labour efficiency of farmers, reduces the costs and time associated with vaccination, improves compliance rates by reducing the errors associated with continuous immunization against different pathogens at similar times, allows the incorporation of new vaccines into the immunization schedule, and reduces the chances of iatrogenic transmission by needles [10]. In this context, the simultaneous administration of PRRSV MLV vaccine and several others has been recently authorized in swine. One such combined administration protocol concerns a PRRSV MLV vaccine with an inactivated PPV and *E. rhusiopathiae* vaccine*.* Data concerning the immunity afforded by such combined administration is yet to be published. In contrast, this information has been published for other vaccines in piglets [11, 12].

We aimed to assess the safety and long-term immunity afforded by the authorized combined administration of a PRRSV MLV vaccine and an inactivated vaccine against PPV and *E. rhusiopathiae* for their simultaneous use. This mixture was administered simulating the classical approach of vaccination, revaccination, and a recall vaccination four months later. Safety was assessed by evaluating systemic and local reactions and body temperature, as well as PRRSV excretion in oral fluid. The immune response was assessed by measuring the levels of PPV, *E. rhusiopathiae*, and PRRSV-antibodies by ELISA. Viral neutralization tests to evaluate homologous neutralizing antibodies (NAs) and cell-mediated immunity (CMI) against four different PRRSV strains were performed before and after every vaccine administration.

## Methods

### Vaccines and viruses

UNISTRAIN^®^ PRRS (HIPRA) is based on an attenuated PRRSV1 strain (VP-046 BIS; 10^3.5–5.5^ CCID_50_ per dose) diluted in PBS. The inactivated vaccine ERYSENG^®^ PARVO (HIPRA) is based on the inactivated *E. rhusiopathiae* strain R32E11 (ELISA > 3.34 inhibition ELISA _50%_) and the inactivated PPV strain NADL-2 (relative potency ELISA > 1.15). This bivalent vaccine is complemented with aluminium hydroxide, DEAE-dextran, and Ginseng as adjuvants. In 2015, the associated administration of these vaccines was given a positive recommendation by the EMA [13].

**Fig. 1:**
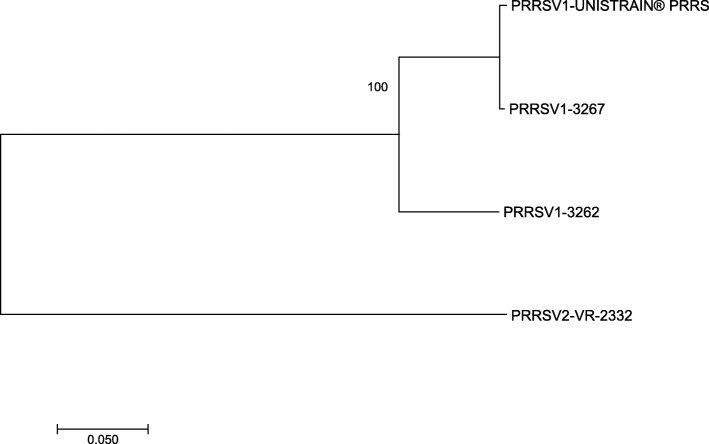
Neighbor-Joining phylogenetic tree among strains based on the complete genome. Confidence of the internal branche – expressed as a value out of 100 -, is based on 1000 bootstrap pseudo-replicates of the pairwised matrix of distances using the gamma Tamura-Nei model

Two PRRSV1 field strains, designated 3262 and 3267, and one PRRSV2 strain (VR-2332), as well as the PRRSV MLV vaccine, were used to evaluate CMI by ELISPOT assay. Both PRRSV1 field strains came from farms showing clinical signs compatible with PRRS; strain 3262 was isolated in 2005 in Spain from a weaner pig that showed respiratory disorders, whereas strain 3267 was isolated in 2006 in Portugal from a boar housed in a farm where sows aborted [14, 15]. VR-2332 is the reference strain of PRRSV2 [16]. All used strains have been entirely sequenced from open reading frame (ORF) 1a to ORF7: genbank accession numbers: JF276431 for 3262, JF276435 for 3267, U87392 for VR-2332, and MK134483 for the PRRSV MLV vaccine. Nucleotide identity per ORF between the vaccine and strains used in the experiments were calculated using MEGA 7 software. Phylogenetic analysis based on the complete genome is shown in Fig. 1. The identity between the PRRSV1 strains and the vaccine was between 83.8% (ORF3 of 3262) and 100% (ORF7 of 3267), whereas the identity values with VR-2332 were much lower (from 58.1 to 72.4%, depending on the ORF).

PRRSV1 viral stocks were prepared and titrations performed in porcine alveolar macrophages (PAM) from PRRSV-free donor animals, whereas the MARC-145 cell line was used for VR-2332. The presence of PRRSV in cell cultures was revealed by immunofluorescence [17]. All assays were performed using a single batch of viral stocks. Viral stocks were free of *porcine circovirus 2* and *Mycoplasma hyopneumoniae*, as demonstrated by PCR [18, 19].

### Animals and experimental design

Ten six-month-old PRRS-naïve healthy gilts were obtained from a PRRSV, PPV, *E. rhusiopathiae*, and *Aujeszky’s disease virus* negative farm. At experimental facilities, animals were ear-tagged and tested to be free of PRRSV, PPV, and *E. rhusiopathiae*-antibodies by commercial ELISAs, detailed below. They were then randomly divided into two groups and placed into two physically isolated boxes: Vaccinated (group V; *n* = 6) and Control (group C; *n* = 4). After one week of acclimatization (day 0), animals in group V were vaccinated intramuscularly with 2 mL freshly mixed UNISTRAIN^®^ PRRS and ERYSENG^®^ PARVO vaccines. They were vaccinated again with the same mixture at days 21 (revaccination) and 147 (recall vaccination). The vaccines were prepared and diluted following the manufacturer’s recommendations. Group C was maintained as a control and gilts were immunized at the same time and via the same route with 2 ml sterile PBS.

### Clinical follow-up and sampling

Vaccine safety following the above-mentioned immunization schedule was assessed by monitoring the systemic and local reactions of the individual animals. Systemic reactions were measured by clinical observation and local reactions at the injection site by visual inspection and palpation (Table 1). In addition, body temperature was recorded immediately before administration of the products and 4 and 24 h later.

**Table 1:** Experimental design

		Day of the experiment										
	0	0 (+ 4 h)	1	21	21 (+ 4 h)	22	28	42	147	147 (+ 4 h)	148	154
Treatment^a^	X			X					X			
Systemic reactions^b^	X	X	X	X	X	X			X	X	X	
Local reactions^c^	X	X	X	X	X	X		X	X	X	X	
Body temperature	X	X	X	X	X	X		X	X	X	X	
Oral Fluid collection	X			X				X	X			X
Blood Sampling	X			X			X	X	X			X

^a^Group V (*n* = 6) was intramuscularly vaccinated with the combined administration of a PRRSV MLV vaccine and an inactivated PPV and *Erysipelothrix rhusiopathiae* vaccine*.* Group C (*n* = 4) was maintained as a control and received PBS

^b^Systemic reactions: (0) active animal that responded to stimuli; (1) poorly active but responded to weak stimuli; (2) did not respond to weak stimuli, but to strong ones; (3) animal did not respond to strong stimuli, such as forcing them to get up or walk

^c^The inoculation site could be distinguished (Y/N); Pain at the injection site (Y/N); Inflammation: (0) no inflammatory reaction, (1) slight, from 0 to 3 cm, (2) moderate, from 3 to 5 cm; and (3) severe, more than 5 cm; Presence of a nodule (Y/N and size in cm)

One sample of oral fluids per group was collected and tested for PRRSV by real-time RT-PCR at days 0, 21, 42, 147, and 154. Blood samples were collected in duplicate (using siliconized and heparinized blood-collecting tubes) and tested for evaluation of the immune response at days 0, 21, 28, 42, 147, and 154. Sera were used for assessment of the presence of specific ELISA antibodies against PRRSV, PPV, and *E. rhusiopathiae*, and homologous NAs against UNISTRAIN^®^ PRRS. Heparinized blood samples were used to perform ELISPOT IFN-γ assays.

### Virological analysis

The presence of PRRSV in oral fluid was determined by real-time RT-PCR [20]. RNA extraction was performed with the RNeasy Mini Kit (Qiagen), according to a modified RNA clean-up protocol. Briefly, 200 μL oral fluid was mixed with 500 μL 70% ethanol, 1 μL carrier RNA, and 700 μL kit-supplied RLT buffer (Qiagen). Then, 700 μL mixed sample was applied to the RNeasy spin column. The kit clean-up protocol for RNA isolation was then followed. Finally, RNA was eluted in 50 μL nuclease-free water and the amount of PRRSV genome determined. The final result is expressed as a Ct value.

### Evaluation of the immune response

#### Humoral response

Sera were tested using commercial ELISA kits for the presence of specific antibodies against *E. rhusiopathiae* (indirect ELISA CIVTEST SUIS SE/MR; HIPRA), PPV (blocking ELISA Ingezim PPV R.11.PPV.K1; Ingenasa), and PRRSV (indirect ELISA IDEXX PRRS X3 Ab Test; IDEXX Laboratories). According to the manufacturers, a sample with a ratio of the sample to positive control (S/P) × 100 value > 40 was considered to be positive in the *E. rhusiopathiae* ELISA. For the PPV ELISA, the antibody titration was determined following the manufacturer’s instructions. A cut-off of 200 was estimated based on results obtained from the negative population prior to immunization as the “mean + 3 times the standard deviation (SD)” and validated by INGENASA (personal communication). A sample with a S/P ratio > 0.4 was considered to be positive by the PRRSV ELISA.

NAs against the PRRSV MLV vaccine were measured by a viral neutralization test following a previously described procedure with minor modifications [21]. Briefly, 50 μl of each serum sample was diluted serially from 1:2 to 1:128 in cell-culture medium. Dilutions were mixed with 50 μl viral suspension containing 200 CCID_50_ of the PRRSV MLV vaccine strain. Virus–serum mixtures were incubated for 1 h at 37 °C and then added to MARC-145 cultures in duplicate (96-well plates) and incubated again for three days at 37 °C in a 5% CO_2_ atmosphere. After incubation, infection of cell cultures was revealed by the addition of an anti-PRRSV antibody (ICH5, Ingenasa) and a fluorescein-labelled anti-mouse IgG antibody (Jackson ImmunoResearch Laboratories). Neutralization was considered to occur when less than 10 fluorescent foci were observed. Neutralization titres were expressed as the log_2_ of the reciprocal of the titre. Neutralization titres ≥1:4 (log_2_ = 2) were considered to be of biological significance. Samples were run in duplicate.

#### IFN-γ specific responses to PRRSV strains

Peripheral blood mononuclear cells (PBMCs) were obtained from heparinized blood samples. PBMCs were used to evaluate the frequency of specific IFN-γ-secreting cells (IFN-γ-SC) against PRRSV1 field strains 3262 and 3267, PRRSV2 prototype VR-2332, and the PRRS MLV vaccine by ELISPOT IFN-γ, as reported elsewhere [22]. The ELISPOT assay was developed using commercial monoclonal antibodies (Porcine IFN-γ P2G10 and biotin P2C11, BD Biosciences Pharmingen) and filter plates (Merck). PBMCs (5 × 10^5^) were stimulated with each PRRSV strain at a multiplicity of infection of 0.1. Unstimulated and PHA-stimulated cells (10 Ug/mL) were used as negative and positive controls, respectively. All tests were performed in duplicate. Reactions were developed by adding 3-amino-9-ethylcarbazole as substrate. PRRSV-specific frequencies of IFN-γ-SC for each strain were calculated by subtracting the counts of the spots in unstimulated wells from those in PRRSV-stimulated wells and expressed as the number of responding cells in 10^6^ PBMCs.

#### Statistical analysis

Statistics were performed using StatsDirect v3.1.8. Results were expressed as the mean ± SD. The non-parametric Mann-Whitney test was used to compare the means between groups, whereas the Friedman test was used to compare the means between sampling days within the same group.

## Results

### Clinical follow-up and body temperature

There were no systemic reactions in any animals and the mean body temperatures at day 0 were lower than 39 °C (group V = 38.62 ± 0.48 °C and group C = 38.67 ± 0.35 °C). Four hours after the first vaccination, the body temperature increased slightly for animals in both groups (group V = 39.17 ± 0.26 °C and group C = 39.25 ± 0.43 °C), returning to below 39 °C at day 1. We observed a similar pattern for subsequent vaccinations, with slight increases in body temperature of between 0.08 and 0.34 °C at 4 h, which then returned to the values registered before revaccination. No significant differences in temperature were recorded between the vaccinated and control groups. Individually, none of the vaccinated pigs showed increases of greater than 1.5 °C over the basal temperature before vaccination/revaccination.

There were mild local reactions in a few animals. The injection site was apparent for three animals in group V, and for one in group C 24 h after the first administration, as well as for two animals from group V and one in group C 4 h after the first revaccination. We observed slight inflammation (classified as category 1) in only one vaccinated animal 4 h after the first vaccination.

### Detection of the PRRSV genome in oral fluids

There was no excretion of PRRSV, examined by genome detection in collective oral fluids, by any control animals throughout the experiment. It was only observed once at day 21 (Ct value = 31.5) in group V.

### Evaluation of the immune response

#### Humoral responses

**Fig. 2:**
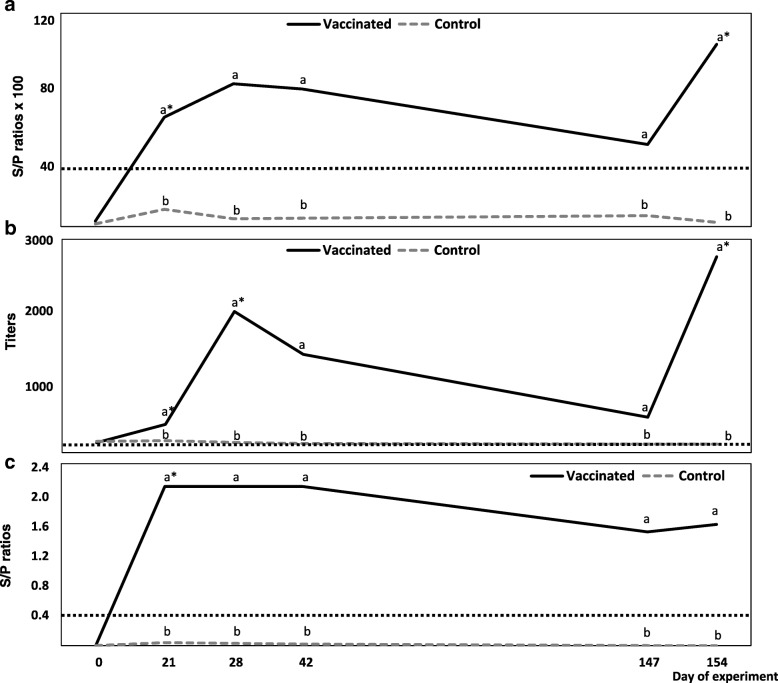
Serological evolution of antibodies against *E. rhusiopathiae* (2**a**), PPV (2**b**) and PRRSV (2**c**) by ELISA. Results are shown as average sample to positive (S/P) ratios in 2**a** and 2**c** and titers in 2**b**. Animals in group V (solid black line) were vaccinated with the combined administration of a PRRSV MLV vaccine and an inactivated PPV and *Erysipelothrix rhusiopathiae* vaccine at days 0, 21 and 147. Animals in group C (dotted grey line) were kept as controls receiving PBS using the same schedule. The cut-off for each ELISA is represented in each figure by a dotted line black. In all cases, different superscript letters (**a**,**b**) indicated statistically significant differences between groups (Kruskal-Wallis test; *p* < 0.05). Statistically significant differences within a group between a given sample and the previous one (Friedman test; *p* < 0.05) were shown as *

The evolution of the humoral response against the three vaccine antigens was measured by ELISA (Fig. 2a-c).

All animals in group C were negative for all ELISAs during the experiment, whereas four of six pigs in group V seroconverted to *E. rhusiopathiae* after the first vaccination and all were positive after re-vaccination. Although the mean S/P ratio × 100 fell from day 28 to day 147, there were no significant differences between samples. Two animals had become negative by day 147, but both once again seroconverted one week later following the recall vaccination. There was a significant boost after the recall vaccination; mean S/P ratio × 100 at day 154 = 102.5 ± 15.6 vs 46.2 ± 15.1 at day 147 (*p* < 0.05) (Fig. 2a).

Analysis of antibodies against PPV showed that all animals in group V already seroconverted after the first administration of the vaccine mixture and remained positive throughout the study period. Mean titres fell from day 28 to day 147, but there were no significant differences. Significant boosts occurred after the first revaccination, as well as after the recall vaccination: mean titres at day 21 = 397.3 ± 121.8 vs 1983.6 ± 637.8 at day 28 (*p* < 0.05) and 496.9 ± 172.7 at day 147 vs 2753.8 ± 1700.3 at day 154 (*p* < 0.05) (Fig. 2b).

At day 21, all vaccinated animals were seropositive for PRRSV by ELISA. The mean S/P ratio remained at a plateau until day 42 and slightly fell afterwards until day 147. No boosts occurred after either revaccination or recall vaccination, although there was a slight increase of the mean S/P ratio in group V after the recall vaccination (Fig. 2c).

Homologous NAs (Table 2) were detected as early as day 21 in all vaccinated animals (individual log_2_ titres from 2 to 3) and remained positive throughout the study. From day 21 onwards, NA titres increased and peaked at day 42 (mean titre ± SD = 4.6 ± 1.2). Remarkably, the titres remained unchanged during the four-month interval (mean titres ± SD = 3.8 ± 0.4 at day 28 vs 3.9 ± 1.3 at day 147). Comparison of the titres showed a significant boost from day 21 to 28 post-vaccination (*p* < 0.05).

**Table 2:** Homologous viral neutralization test (VNT): neutralizing antibodies against the PRRSV MLV vaccine strain

		Day of the experiment					
	Group	0	21	28	42	147	154
Proportion of positive pigs Homologous VNT (log_2_ titer)^a^ Rank	V	0/6	6/6	6/6	6/6	6/6	6/6
			2.5 ± 0.5	3.8 ± 0.4^*^	4.6 ± 1.2	3.9 ± 1.3	4.0 ± 0.4
			(2–3)	(3–4)	(3–6.6)	(2–6.0)	(3.6–4.6)
	C	0/4	0/4	0/4	0/4	0/4	0/4

Results are expressed as the mean ± standard deviation

^a^Neutralization titres ≥1:4 (log_2_ = 2) were considered to be of biological significance

^*^Statistically significant differences between a given sample and the previous one (*p* < 0.05) (Friedman test)

#### Evolution of homologous and heterologous PRRSV-specific IFN-γ-SC

**Fig. 3:**
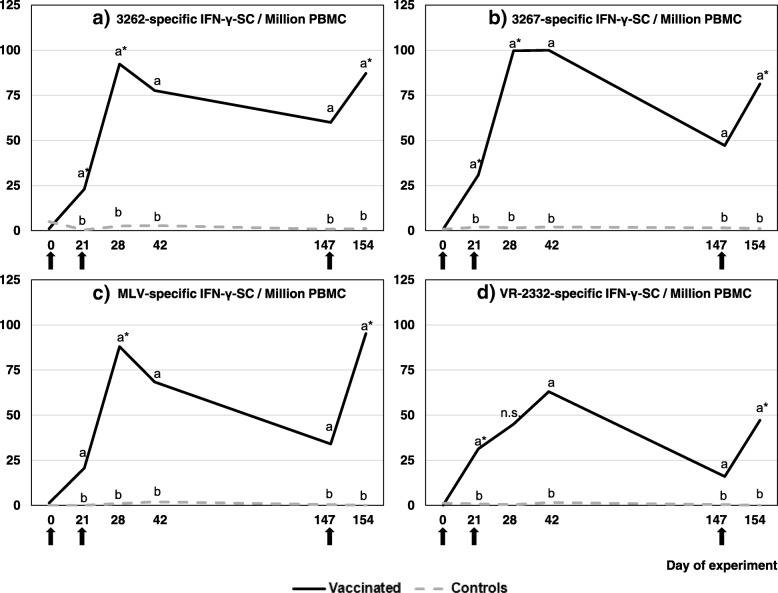
Comparison among groups of PRRSV-specific cell-mediated immune responses (IFN-γ ELISPOT) for a given strain. Peripheral blood mononuclear cells (PBMC) were stimulated with either of the PRRSV strains: Fig. 3**a** (PRRSV1 3262); Fig. 3**b** (PRRSV1 3267), Fig. 3**c** (MLV PRRSV1) and Fig. 3**d** (PRRSV2 VR-2332). In all figures, solid black line corresponds to vaccinated animals: (↑) vaccinated with the combined administration of a PRRSV MLV vaccine and an inactivated PPV and *Erysipelothrix rhusiopathiae* vaccine at days 0, 21 and 147 of the experiment. Dotted grey line correspond to controls. Results are shown as average frequencies of virus-specific IFN-γ secreting cells per million of PBMC. Different superscript letters (**a**,**b**) indicate statistically significant differences between groups (Kruskal-Wallis test; *p* < 0.05). n.s. = non-significant. * Statistically significant differences within a strain between a given sample and the previous one (Friedman test; *p* < 0.05)

We followed the evolution of the PRRSV-specific IFN-γ-SC for each strain in both groups (Fig. 3a-d). The mean for group C remained below three PRRSV-specific IFN-γ-SC/million PBMCs for all strains. The vaccinated group already showed significantly higher means at day 21 for all strains (*p* < 0.05). The mean of group V then remained significantly higher than that of group C throughout the experiment, except for VR-2332 at day 28.

More specifically, stimulation of PBMCs from vaccinated pigs with PRRSV1 field strains resulted in peak frequencies for 3262-specific IFN-γ-SC at day 28 (92.3 ± 99.4), whereas a peak for 3267 occurred at day 42 (100.0 ± 131.2), although it already reached the same value at day 28 (99.7 ± 122.8). Stimulation with the PRRSV MLV strain vaccine (called a homologous response) resulted in the highest mean at day 154 (95.2 ± 61.2), although a similar value was already reached at day 28 (88.0 ± 84.6). Stimulation with the PRRSV2 prototype strain VR-2332 resulted in a lower peak than for the other strains (63.0 ± 60.0 at day 42).

Evaluation of the evolution of the responses showed a clear decrease between day 42 and day 147. However, there were no significant differences in responses between these days for any of the assayed strains. On the contrary, the mean of PRRSV-specific IFN-γ-SC increased one week after each re-administration of the vaccine. Indeed, we observed boosts for all PRRSV1 strains after revaccination and recall vaccination (*p* < 0.05), whereas a significant boost only occurred after recall vaccination for VR-2332 (*p* < 0.05).

## Discussion

We evaluated co-immunization with the licenced combined administration of a PRRSV MLV vaccine and an inactivated PPV and *E. rhusiopathiae* vaccine for their simultaneous use following a common administration schedule used in the field. It is conceivable that the interaction of antigens, excipients, and adjuvants in the formulation of a combined administration of vaccines could cause negative interference among them [10]. This possibility thus merited investigation.

At least three main issues must be addressed when two or more vaccines are combined and injected simultaneously: 1) the stability of the antigens, 2) the safety of the mixture, and 3) the immunity afforded against each antigen.

The stability of the antigens in the combined administration of vaccines acquires greater relevance when an attenuated virus is included in the mixture. It has been demonstrated that specific components of a mixture can sometimes inactivate the virus, which could lose efficacy in such mixtures [23]. This aspect could be worrisome, given that PRRSV is generally found in low amounts in MLV vaccines [24]. Moreover, mass vaccination of sows is very time consuming, even more so in medium/large-scale farms. Thus, product stability is crucial to guarantee that all animals are correctly vaccinated. PRRSV MLV strain used in our study has been guaranteed to remain viable for at least two hours after mixing with the PPV and *E. rhusiopathiae* vaccine [25]. Moreover, the virus was detected in the oral fluids of vaccinated animals, showing that it was replicating and shedding, as expected for a PRRSV MLV vaccine injected alone [24].

Concerning safety, adjuvants could increase the adverse effects of vaccines. Saponins, aluminium compounds, and lipopolysaccharide or derived products may enhance side effects, such as inflammation or granulomatous reactions, and non-specific systemic reactions, such as fever or anorexia [26]. Here, we administered three different adjuvants, together with all the excipients, in just one injection during successive revaccinations. However, this combined administration of vaccines was safe in terms of local and systemic reactions and body temperature, meaning that the mixture is safe, even under such conditions.

The mixture provided good immunity, as the ELISA results demonstrated significant boosts after revaccination for PPV and after recall vaccination for both PPV and *E. rhusiopathiae*. Although the values for both ELISAs fell during the four-month interval, the differences were not significant. From a practical point of view, these results show that an immunization schedule based on a four-month interval can maintain the humoral response of breeding herds against these two antigens. It is possible that aluminium compounds included in the bivalent vaccine enhanced humoral responses by themselves, due to the depot effect and an increase in antigen uptake by antigen presenting cells [27]; however, the possibility of a synergetic effect of the adjuvants should not be discarded [28]. In contrast to PPV and *E. rhusiopathiae*, no boosts by PRRSV ELISA were observed, despite the adjuvants. This is in accordance with other studies in which detectable boosts, in terms of antibodies measured by commercial ELISAs after PRRSV re-infection or revaccination, were not observed [15, 29]. It is important to note that these PRRSV-specific antibodies are usually measured for diagnostic purposes and monitoring herds and are not related to protection [30]. Contrary to the ELISA results, we observed a significant boost of NAs after revaccination. It is possible that differences observed between antibodies against PRRSV measured by ELISA and NAs could be due by the fact that a booster effect may only exist against some viral antigens not included in the coating for the ELISA [15, 29].

It is well-known that PRRSV generates an immune response characterized by the weak and delayed production of NAs and CMI development, which does not fit in classical vaccinology [24, 30–33]. Moreover, the role of both NAs and CMI during the clearance of the virus and protection is a recurring topic of discussion in PRRSV immunology [33]; it has been suggested that NAs may play a role in protection, whereas CMI might be related to clearance and protection in the absence of NAs [15, 33]. Nevertheless, both NAs and IFN-γ are the most highly studied immune mechanisms of protection against PRRSV, as they seem to play an important role [33]. We detected PRRSV-specific NAs in all vaccinated animals as soon as 21 days post-vaccination. NAs generally appear late for both PRRSV field-strains and PRRSV MLV [30, 33]. The reason for the poor NA response against PRRSV is unclear, but glycosylation within epitopes or flanking neutralising epitopes and the presence of immunodominant decoy epitopes have been postulated [33]. During the vaccination period, some animals reached NA titres that could be considered to be high relative to those of other studies [33].

We used four strains of diverse and unrelated nature to widen the focus of CMI against PRRSV. As expected, the lowest CMI responses occurred for the PRRSV2 strain. We observed similar patterns for PRRSV1 strains and differences among strains on a given day were not seen (data not shown), despite the marked genetic differences and different immunological properties [14, 15, 34]. CMI boosts associated with repeated administrations of PRRSV MLV are unusual. It has been hypothesized that repeated homologous immunization with PRRSV MLVs induces very limited responses [9, 29], due to the dysregulation caused by the virus [7], or even possibly a state of anergy [8]. However, our results clearly show that this phenomenon did not occur with the combined administration of the vaccines. In contrast, this mixture induced long-lasting immunity using the four-month interval schedule, characterized by boosts after each administration, at least for the PRRSV1 strains used.

One of the main goals of vaccine adjuvants is to increase the immune response of poor antigens [28] or antigens linked with immune dysregulation, such as PRRSV. Indeed, it was previously assumed that the co-administration of adjuvants with PRRSV MLVs would fail to enhance immune responses; however, this notion was based on just one adjuvant [35]. Evidently, this assertion may not always be true as the nature and mechanisms of action of adjuvants are highly diverse [27]. More recently, several groups have demonstrated that specific adjuvants can result in a large improvement in virus-based vaccines against PRRSV [32, 36, 37]. In our study, the causes behind the similarities between CMI responses against different strains and the boosts detected are unknown, as well as the early stimulation of NAs, and merit further investigation. However, it is possible that the adjuvants in the mixture of the vaccines could have somehow been related with these observations. In this context, ginsenosides, a type of saponin found in ginseng, have proven to be a safe adjuvant to enhance CMI responses, since high amounts of IFN-γ have been detected against different antigens in several species [38–40]. On the contrary, aluminium compounds show a limited ability to enhance CMI responses [27]. Thus, ginseng improves the immune response to vaccines containing aluminium as a co-adjuvant [41]. It cannot be ruled out that the CMI responses observed in our study may have been due to a synergic effect of the adjuvants, as suggested for humoral responses [41, 42].

Vaccination strategies on farms should be established to achieve an adequate level of immunity against etiological agents. The vaccination schedule used for gilts associated with the combined administration of vaccines against PRRSV, PPV, and *E. rhusiopathiae* induced an immune response as early as day 21 and long-lasting immunity for four months in the vaccinated animals. After this period, the recall vaccination of sows boosted the immune response. These results support the validity of this vaccination schedule, which is commonly implemented for gilts and sows in the field to sustain their immunity.

## Conclusions

For the first time, long-term immune responses against three swine pathogens using the combined administration of two vaccines for their simultaneous use were evaluated. A vaccination schedule based on two administrations in gilts and at four-month intervals in sows consisting of the combined administration of two vaccines, showed it:

- To be safe
- To induce long-lasting immunity against PRRSV, PPV, and *E. rhusiopathiae*.
- To boost humoral responses against PPV and *E. rhusiopathiae.*
- To boost CMI after each administration against genetically and immunologically diverse PRRSV strains, contrary to what usually happens following continued administration of PRRSV MLVs.
- To induce a homologous NA response by day 21, which remained constant thereafter.

Our results show that the combined administration of vaccines, following a vaccination schedule commonly used in the field, may be helpful in developing, maintaining, or even increasing immune responses against PRRSV, PPV, and *E. rhusiopathiae*.

## Abbreviations

CCID_50_: Cell culture infectious dose 50%
CMI: Cell-mediated immunity
ELISA: Enzyme-linked immunosorbent assay
ELISPOT: Enzyme-linked immuno-spot assay
IFN-γ: Interferon gamma
MLV: Modified live virus
MOI: Multiplicity of infection
NA: Neutralizing antibody
NS: Nonsignificant
ORF: Open reading frame
PAM: Porcine alveolar macrophage
PBMC: Peripheral blood mononuclear cells
PBS: Phosphate-buffered saline
PCR: Polymerase chain reaction
PHA: Phytohemagglutinin
PPV: *Porcine parvovirus*
PRRSV: *Porcine reproductive and respiratory syndrome virus*
RT-PCR: Reverse-transcription polymerase chain reaction
S/P: Sample/positive
SC: Secreting cells
SD: Standard deviation
